# Reconsidering the management of patients with cancer with viral hepatitis in the era of immunotherapy

**DOI:** 10.1136/jitc-2020-000943

**Published:** 2020-10-16

**Authors:** Dimitrios C Ziogas, Frosso Kostantinou, Evangelos Cholongitas, Amalia Anastasopoulou, Panagiotis Diamantopoulos, John Haanen, Helen Gogas

**Affiliations:** 1 First Department of Medicine, National and Kapodistrian University of Athens School of Medicine, Athens, Greece; 2 Division of Medical Oncology, The Netherlands Cancer Institute, Amsterdam, The Netherlands

**Keywords:** immunotherapy, review

## Abstract

In the evolving immune-oncology landscape, numerous patients with cancer are constantly treated with immune checkpoint inhibitors (ICPIs) but among them, only sporadic cases with pre-existing hepatitis B virus (HBV) and hepatitis C virus (HCV) are recorded. Despite the global dissemination of HBV and HCV infections, viral hepatitis-infected patients with cancer were traditionally excluded from ICPIs containing trials and current evidence is particularly limited in case reports, retrospective cohort studies and in few clinical trials on advanced hepatocellular carcinoma. Thus, many concerns still remain about the overall oncological management of this special subpopulation, including questions about the efficacy, toxicity and reactivation risks induced by ICPIs. Here, we examine the natural course of both HBV and HCV in cancer environment, review the latest antiviral guidelines for patients undergoing systematic cancer therapies, estimating treatment-related immunosuppression and relocate immunotherapy in this therapeutic panel. Among the ICPIs-treated cases with prior viral hepatitis, we focus further on those experienced HBV or HCV reactivation and discuss their host, tumor and serological risk factors, their antiviral and immunological management as well as their hepatitis and tumor outcome. Based on a low level of evidence, immunotherapy in these specific cancer cases seems to be associated with no inferior efficacy and with a relevantly low reactivation rate. However, hepatitis reactivation and subsequent irreversible complications appeared to have poor response to deferred antiviral treatment. While, the prophylactic use of modern antiviral drugs could eliminate or diminish up front the viral load in most cases, leading to cure or long-term hepatitis control. Taking together the clinical significance of preventive therapy, the low but existing reactivation risk and the potential immune-related hepatotoxicity, a comprehensive baseline assessment of liver status, including viral hepatitis screening, before the onset of immunotherapy should be suggested as a reasonable and maybe cost-effective strategy but the decision to administer ICPIs and the necessity of prophylaxis should always be weighed at a multidisciplinary level and be individualized in each case, up to be established by future clinical trials.

## Introduction

Immune checkpoint inhibitors (ICPIs) have drastically transformed clinical cancer care and continue to expand further their therapeutic indications.^1^ Following these evolving immune-oncology guidelines, an increasing number of patients with cancer is exponentially exposed to ICPIs, but among them, only sporadic cases with hepatitis B virus (HBV) or hepatitis C virus (HCV) infections have been recorded.^2^ Despite that both hepatotropic viruses infect millions of people worldwide,^3 4^ clinical experience of immunotherapy in this special population of viral hepatitis-infected patients with cancer is limited to case reports,^5–8^ retrospective cohort studies^9^ or few clinical trials in advanced hepatocellular carcinoma (HCC).^10–12^ In the majority of pivotal trials that led to initial immunotherapy approvals, patients with prior infections with HBV or HCV were traditionally excluded because of immune-related concerns about efficacy, toxicity and reactivation risks.^2 13–15^

In support, some recent studies showed that unbalancing the immune system and releasing the T cell-mediated cytotoxicity via checkpoint inhibition could cause reactivation of HBV, HCV or other pre-existing chronic infections such as tuberculosis.^9 16^ Except for the potential reactivation risk, the hepatitis-induced liver damage together with the liver involvement of metastatic cancer and the immune-related hepatotoxicity may delay or even cause discontinuation of anticancer therapy, impacting further on the disease outcome. In parallel, chronic viral hepatitis increases the risk of cirrhosis and HCC development, but also the risk of several extrahepatic malignancies.^17^ Considering chronic viral hepatitis as a continuing global health hazard, the United Nations and the WHO adopted the elimination of HBV and HCV in their major goals for the close future.^18^

Based on the current evidence, we examine here the hepatitis behavior in cancer environment, estimate the level of immunosuppression of administered cancer therapies and evaluate the oncological implications of both HBV and HCV on treatment and tumor outcome. Among the identified ICPIs-treated cases with HBV or HCV infections, this review focuses further on those patients experiencing hepatitis reactivation, and tries to evaluate the rate of viral exacerbation and to recognize any potential risk factors, therapy-related or not. In relation to estimated efficacy and safety of ICPIs in this non-trial population, the guidelines for screening, monitoring, immunological management as well as the recommendations for antiviral prophylaxis and on-demand treatment are thoroughly discussed and reconsidered.

## Natural history of HBV or HCV infections

The biological course of HBV or HCV infections is determined by the interplay between viral replication and host’s immune response. Both HBV and HCV are small, enveloped viruses containing circular double-stranded DNA and single-stranded RNA, respectively, that are transmitted by blood or bodily fluids and cause mainly hepatocellular injury. Once hepatocytes are infected, innate and adaptive immune systems are activated secondary to viral immunotropism. More specifically, secreted interferons by natural killer cells emerge the T cell-mediated response; CD4+ T cells are involved in the production of neutralizing antibodies by B cells, whereas CD8+ T cells eliminate virus-infected hepatocytes via cytotoxicity, inducing liver immunopathology. Many hypothetic mechanisms of hepatitis reactivation during checkpoint inhibition have been suggested but none of them has undoubtedly been established. The blockade of programmed cell death-1/programmed cell death 1 ligand (PD-1/PD-L1) axis may restore HBV-specific CD8+ T cells that overexpressed PD-1 due to chronic viremia,^19 20^ leading to overwhelming liver damage and further release of previously latent viruses into circulation.^21^ Moreover, the inhibition of PD-1/PD-L1 engagement may promote the proliferation of T regulatory cells, increasing immunosuppression and weakening the epistasis of chronic HBV infection.^22^ On the other hand, the only clinical trial investigating the use of anti-PD-1 antibody in patients with chronic HCV infection showed that some patients have persistent suppression of HCV replication, but only 12% had a sustained and durable reduction in HCV RNA.^23^ More research is needed to reveal the underlying immune-mediated mechanisms of viral reactivation that are impaired by anti-PD-1 therapy.

In immunocompetent individuals, most primary infections are self-limited and spontaneously resolved after their acute phase, establishing protective antibodies. However, in 60%–85% of exposed patients, a chronic setting of hepatitis is developed due to weaker innate and adaptive immune responses. The viral load is not sustainably cleared and may persist in hepatocytes for >6 months, even after serological recovery. The chronicity of hepatitis places these patients at later risks of cirrhosis and hepatocarcinogenesis. Chronic HBV causes approximately 60% of HCC worldwide, whereas 20% and 50% of cases with HCC in Asia and in the USA, respectively, are associated with chronic HCV.^17 24^ Except for HCC, chronic hepatitis is also related to hematologic malignancies, including B cell non-Hodgkin's lymphoma (B-NHL)^25^ and to some second primary solid malignancies including colorectal cancer,^26^ intrahepatic cholangiocarcinoma,^25^ head and neck, renal and pancreatic cancers.^25 27 28^

In chronic hepatitis setting, any additional reconstitution of immune control after immunosuppressive medications could trigger viral reactivation. Similarly to acute infection, hepatitis reactivation could be presented with a variety of non-specific clinical manifestations, ranging from asymptomatic flare mainly of alanine aminotransferase (ALT) or aspartate aminotransferase (AST) to fatal liver damage.^29 30^ The hepatitis flare, defined as an increase in ALT to ≥3 times the upper limit of normal, is typically preceded by the the rise in viral DNA/RNA by 2–3 weeks.^31^ Regarding HBV reactivation (HBVr), its diagnosis was initially based on the serological upregulation of hepatitis B surface antigen (HBsAg) and anti-HBsAg titers compared with baseline measurements. However, the addition of quantitative HBV DNA assay made the definition of HBVr more reliable but more complicated and heterogeneous among studies and hepatology societies. For instance, the American Association for the Study of Liver Diseases (AASLD) defines HBVr as the elevation of HBV DNA compared with baseline in combination with seroconversion to HBsAg(+) for patients with HBsAg(−), anti-HBc(+)^32^; the European Association for the Study of the Liver (EASL) gives no clear definition of HBVr^33^; while the Asian Pacific Association for the Study of the Liver outlines HBVr as a significant increase of HBV DNA from baseline levels or the detection of ≥20 000 IU/mL in previously undetectable viral burden.^34^ In clinical practice, screening and close monitoring of serum ALT and of HBV DNA can lead to early diagnosis and management of HBVr, especially in patients with high-risk factors for HBV infection, independent of the planned systemic cancer therapy and in patients anticipating high-risk HBVr anticancer therapy, independent of HBV infection risk.^31 35^ In hepatitis C, laboratory testing for anti-HCV antibody is not enough to distinguish between acute and chronic setting, and HCV reactivation (HCVr) is diagnosed only by an increase in HCV RNA ≥1 log IU/mL over baseline.^36^ In this clinical scenario, testing of liver functions and surveillance of anti-HCV and of viral load levels can ensure early identification of HCV infection or HCVr.

## Prevalence of viral hepatitis in cancer and risk factors of reactivation

The global numbers of viral hepatitis are growing with threatening pattern. Approximately 2×10^9^ persons have been previously infected with HBV and clinically resolved their infection, HBsAg(−)/anti-HBc(+)^3^; while more than 250×10^6^ developed chronic infection, HBsAg(+)/anti-HBc(+).^3^ On the other hand, 71 million people are chronically infected by hepatitis C, and of these, 80% are undiagnosed, mostly because they are asymptomatic.^4 37^ Based on data from Centers for Disease Control and Prevention (CDCP) on the US population, the overall prevalence rate of past HBV was 4.7%, of chronic HBV 0.3%, and of HCV 1.3%.^38–40^ However, both HBV and HCV infections were found more prevalent among older persons and particularly among those with cancer.^41^ A recent multicenter prospective cohort study of more than 3000 patients with newly diagnosed cancer calculated an observed infection rate of 6.5% (95% CI: 5.6% to 7.4%) for previous HBV infection, 0.6% (95% CI: 0.4% to 1.0%) for chronic HBV, and 2.4% (95% CI: 1.9% to 3.0%) for HCV.^2^Among those, a substantial proportion was unaware of their viral status at the time of cancer diagnosis (87.3% with previous HBV, 42.1% with chronic HBV, 31.0% with HCV), while many had no identifiable risk factors (27.4% with previous HBV, 21.1% with chronic HBV, 32.4% with HCV).^2^ Cancers of liver, gastrointestinal tract, head and neck, lung and prostate had the highest prevalence of viral infection but frequencies differed significantly within cancer by the type of viral infection. Because of viral status, the therapeutic decision was changed in only 8.0% of patients, independently of hepatitis type.^2^

According to Loomba and Liang, the main risk factors for HBVr can be categorized as virus-related, host-related, and medication-related factors.^29^ High-risk viral factors for HBVr include markers that characterize high baseline viral burden: detectable HBV DNA,^42^ mutations of HBsAg,^43^ HBeAg(+), chronic HBV infection with HBsAg(+), anti-HBc(+),^42^ specific HBV genotype^43 44^ and coinfection with other viruses, such as HCV or hepatitis D.^29 31 43^ Among these viral risk factors, detectable levels of HBV DNA were recognized to be the most significant determinant since 37.8% of patients with cancer with detectable levels experienced HBVr when undergoing chemotherapy^42^; while patients with chronic HBV infection (HBsAg(+)/anti-HBc(+)) have up to an eightfold increased risk of HBVr compared with patients with resolved infection (HBsAg(−)/anti-HBc(+)).^45^ Interestingly, a recent meta-analysis showed that HBVr occurs more frequently in HBV/HCV coinfected patients undergoing anti-HCV treatment with direct-acting antivirals (DAAs), compared with not coinfected patients.^46^ Other host factors associated with high risk of HBVr include older age, male sex, cirrhosis and underlying disease inducing or requiring immunosuppression (eg, lymphoma, solid tumors, rheumatoid arthritis).^47^

In cancer setting, HBVr can spontaneously occur as a complication during progression,^48^ but is more commonly induced by the immunosuppression of administered therapies (eg, monoclonal antibodies, cytotoxic chemotherapy or transplantation) in patients with HBsAg(+) or previous HBV exposure (HBsAg(−)/anti-HBc(+)). Previous studies have estimated that HBVr from anticancer therapies occurred in 41%–53% of patients with HBsAg(+)/anti-HBc(+) and in 8%–18%of patients with HBsAg(−)/anti-HBc(+).^49 50^ These rates of HBVr are significantly higher compared with those observed under antirheumatic immunosuppressive regimens, 12.3% in patients with HBsAg(+)/anti-HBc(+) and 1.7% in patients with HBsAg(−)/anti-HBc(+).^51 52^ The American Gastroenterology Association (AGA)^53 54^ and the AASLD^32^ have categorized the individual risk of certain immunosuppressive agents for HBVr coestimating the serological profile of treated patients, with few differences for HBsAg(−) cases. Integrating both stratifications, patients could be discerned into the following HBVr risk groups, presented in table 1. As new immune-modulatory agents enter into the clinical arena, their impact on HBV or HCV infection needs to be continuously reconsidered. The last European Society of Medical Oncology (ESMO) review in 2016 for medication-related factors associated with HBVr recognized that most oncological drugs frequently used could induce HBVr in patients with HBsAg(+) and recommended HBV screening before any systemic anticancer treatment initiation.^55^ In the same study, anthracyclines, vinca-alkaloids, methotrexate, cyclophosphamide, etoposide and everolimus were associated with the highest frequency of HBVr. However, up to the review publication date,^54^ no case of HBVr was documented with ICPIs. Voican *et al* concluded that pre-emptive antiviral treatment could reduce the adverse effects of HBVr and prevent chemotherapy interruption.^55^ In the first retrospective study of 114 patients with HBsAg(+) with cancer undergoing PD-1 inhibition (male 79% and median age 46 years), only 6 (5.3%) patients developed HBVr and these are thoroughly discussed below.^9^ In this study, Zhang *et al* noticed that the lack of antiviral prophylaxis was the only significant risk factor for HBVr (OR=17.50, 95% CI: 1.95% to 157.07%, p=0.004).^9^ Patients under antiviral preventive therapy had significant lower rate of HBVr and hepatitis flare compared with those without prophylaxis (1.2% vs 17.2%, p=0.004 and 1.2% vs 13.8%, p=0.019). Without reaching statistical significance, patients with HBeAg(+) appeared to have increased risk of HBVr (OR=6.25, 95% CI:0.99 to 39.50, p=0.086), while patients with HCC had higher risk of aminotransferase elevation than those with other cancer types (OR=2.52, 95% CI: 1.04 to 6.12, p=0.038) but no higher HBVr risk. Baseline HBV DNA levels or immunotherapy options were not associated with HBVr.

**Table 1:** HBVr risk groups in patients with cancer according to their anticipating immunosuppressive treatment

Risk group	HBVr rate (%)	Hepatitis condition	Anti-HBc Status	HBsAg status	Anticipating immunosuppressive anticancer treatments
Very high risk	>20	Chronic infection	Anti-HBc(+)	HBsAg(+)	Anti-CD20 therapy (ie, rituximab, ofatumumab, obinutuzumab) or hematopoietic SCT.
High risk	11–20	Chronic infection	Anti-HBc(+)	HBsAg(+)	High-dose steroids (ie, ≥20 mg/day for at least 4 weeks); anthracycline derivatives such as doxorubicin and epirubicin; or the anti-CD52 agent, alemtuzumab.
Moderate risk	1–10	Chronic infection	Anti-HBc(+)	HBsAg(+)	Cytotoxic chemotherapy without steroids; anti-TNF therapy; cytokine and integrin inhibitors, tyrosine kinase inhibitors, or anti-rejection therapy for solid organ transplants.
		Clinically resolved infection	Anti-HBc(+)	HBsAg(−)	Anti-CD20 therapy or hematopoietic SCT.
Low risk	<1	Chronic infection	Anti-HBc(+)	HBsAg(+)	Methotrexate or azathioprine and any dose of steroids lasting less than a week or low-dose (eg, <10 mg prednisone daily) lasting greater than or equal to 4 weeks.
		Clinically resolved infection	Anti-HBc(+)	HBsAg(−)	High-dose glucocorticoids (eg, ≥20 mg/day) or the anti-CD52 agent, alemtuzumab.
Very low risk	Rarely occurs	Clinically resolved infection	Anti-HBc(+)	HBsAg(−)	Cytotoxic chemotherapy without steroids, anti-TNF therapy, methotrexate or azathioprine.
Uncertain risk		Clinically resolved infection	Anti-HBc(+)	HBsAg(−)	Solid organ transplant influenced by the type and the degree of used immunosuppressive therapy.

anti-HBc, anti-hepatitis B core antibody; HBsAg, hepatitis B surface antigen; HBV, hepatitis B virus; HBVr, HBV reactivation; SCT, stem cell transplantation; TNF, tumor necrosis factor.

Nevertheless, type of malignancy may also play a role in HBVr. In a retrospective study of patients with HBsAg(+) with solid tumors or hematological malignancies who underwent chemotherapy without antiviral therapy, the incidence of severe acute HBV exacerbation was higher in patients with hematologic malignancies than in those with solid tumors (25.0% vs 4.3%, p=0.005) and in cases receiving rituximab-based chemotherapy than in those receiving non-rituximab-based chemotherapy (40.0% vs 4.1%, p=0.001).^56^ Among the patients with solid tumors, the observed rates of HBVr in HCC, colorectal cancer, lung cancer, breast cancer, gynecological cancer, urological tract cancer, head and neck squamous cell cancer (HNSCC) and other solid malignancies were 2.3%, 4.0%, 7.1%, 9.0%, 16.7%, 6.7%, 0% and 0%, respectively.^56^ Another recent study on patients with leukemia with prior resolved HBV infection recognized that the incidences of HBVr were higher in patients receiving hematopoietic stem cell transplantation (SCT, 5.7%) compared with those receiving chemotherapy (2.2%); while given that anti-HBe(−), high anti-HBs or low anti-HBc levels at baseline were associated with a low risk of HBVr.^57^

In an effort to determine the incidence and prognosis of HCVr during anticancer treatment, an observational study from MD Anderson Cancer Center estimated an overall reactivation rate of 23%, ranging from 36% in patients with hematologic malignancies to 10% in patients with solid tumors.^36^ In univariate analysis, HCVr occurred more frequently in patients with prolonged lymphopenia (median, 95 vs 22 days, p=0.01) and in cases receiving rituximab (44% vs 9%; p<0.0001), bendamustine (22% vs 0%; p<0.001), high-dose steroids (57% vs 21%; p=0.001), or purine analogs (22% vs 5%; p=0.02). In multivariable analysis, the effect of rituximab (OR=9.52; p=0.001), and high-dose steroids (OR=5.05; p=0.01) retained clinical significance. Among the 23 patients with HCVr, only 10 (43%) had hepatitis flare and no liver failure or liver-related death was recorded within 36 weeks after initiation of cancer treatment.^36^ Patients with HCVr had an unremarkable clinical course; however, 26% (6 of 23) of them required unanticipated discontinuation or dose reduction of cancer treatment. These findings support that early identification and treatment of chronic HCV infection prevent complications by viral reactivation, avoiding in parallel major changes in the cancer therapeutic plan.

## Current evidence on HBV or HCVr under ICPI treatment

From the beginning of immunotherapy era up to now, 10 isolated incidents of HBVr after treatment with ICPIs have been described in four case reports^5–7^ and in one retrospective cohort study.^9^ At baseline, eight of them were HBsAg(+)/ antiHBc(+), one was HBsAg(−)/antiHBc(+) and one had no viral work-up due to normal liver function tests. The patients and tumor characteristics of these 10 cases, baseline viral profile, administered immunotherapy and antiviral approach as well as hepatitis outcomes are presented in table 2. The underlying tumor types of these identified patients were lung adenocarcinoma (n=2), nasopharyngeal carcinoma (n=2), melanoma (n=2), HCC (n=1), clear-cell renal cell carcinoma (n=1), HNSCC (n=1) and soft tissue sarcoma (n=1). These 10 patients with HBVr were treated with a PD-1/PD-L1 blocking antibody and one had previously received ipilimumab (anti-cytotoxic T-lymphocyte-associated protein 4 (CTLA-4)) for four cycles. HBVr developed within a median of 14 weeks (range, 3–40 weeks) after the initiation of immunotherapy. Nine episodes of HBVr were documented during immunotherapy, while hepatitis B was reactivated in the last case 6 weeks after the discontinuation of ICPI. At baseline, eight patients had undetectable HBV DNA (<20 IU/mL) and two no measurable viral load. At reactivation, the median HBV DNA level was 2055 × 10^3^ IU/mL (range, 244.259 to >170,000 × 10^3^ IU/mL), based on the available data. Eight of 10 patients were diagnosed with HBV-related flare with a median peak ALT of 289.6 U/L (range, 19–994 U/L) and a median peak of AST 485.5 U/L (range, 28–845 U/L). One patient exhibited an increase in HBV DNA level without ALT elevation. Two patients were receiving prophylactic entecavir before the initiation of immunotherapy; one was receiving abacavir due to HBV/HIV coinfection and had recently been vaccinated twice for HBV while the remaining seven were not under antiviral pre-emptive therapy. In the HBV/HIV coinfected case, abacavir switched to tenofovir disoproxil fumarate (TDF) respecting the patient’s decision to avoid adding a third medication into his antiretroviral therapy. After the occurrence of HBVr, four of seven patients without antiviral prophylaxis were treated with entecavir, one patient was treated with tenofovir and administration of steroids for potential autoimmune hepatitis, one patient was treated with TDF concurrently with nivolumab since aminotransferases were already elevated from prior treatment with ipilimumab; while the patient without ALT elevation did not receive any antiviral treatment but the HBV DNA spontaneously turned to undetectable 5 weeks later. For the one case with prophylactic entecavir, antiviral treatment was intensified with the addition of TDF at the time of reactivation. Due to HBVr, six patients disrupted their immunotherapy, including one discontinuation and five treatment delays. Three cases continued uninterruptedly their treatment with ICPIs and for the last one no information was available about her immunological management. For the entire cohort, HBVr has resolved and all the 10 patients achieved undetectable or diminished HBV DNA levels after a median of 6.5 weeks (range 1–40 weeks). No HBV-related fatal events occurred up to publication of these case reports. Kothapalli and Khattak^58^ treated five patients with past HBV infection (HBsAg(−)/anti-HBc(+), one had also chronic HCV infection) with ICPIs for either metastatic

**Table 2:** Published cases of HBV and HCVr in patients with cancer treated with immunotherapy

A/a	First author, year	Age (gender)	Cancer type	Type of ICPI	Baseline HBV DNA (IU/mL)	Baseline hepatitis panel	Baseline AST/ALT	Antiviral prophylaxis	Duration on ICPI at reactivation	Reactivation HBV DNA (IU/mL) and hepatitis panel	Reactivation AST/ALT (U/L)	Management of ICPI	Antiviral treatment	Time for undetectable HBV DNA (weeks)	Time for ALT (ULN:56 U/L) and AST (ULN:40 U/L) recovery (weeks)
1	Lake, 2017^5^	72 (M)	NSCLC stage IIIa TTF-1 (+)	Nivolumab (April 2016)	<20	Anti-HBc: (+) HBsAg: (−) HIV infection (ART: dolutegravir 50 mg once daily, abacavir 600 mg once daily since February 2016)	Normal	Two doses of HBV vaccine (July 2014 and November 2015)	4 weeks	HBV DNA>170,000,000	AST: 301 U/L ALT: 332 U/L	Delayed for 3 months	Switched from abacavir to TDF but patient declined adding of third drug to ART	Not achieved on follow-up (16 weeks) but significantly decreasing	ALT/AST: normalized after 12 and 16 weeks, respectively
2	Pandey, 2018^6^	51 (M)	NSCLC stage IV TTF-1 (+)	Pembrolizumab	No baseline hepatitis panel	No baseline hepatitis panel	Normal	None	4 weeks	HBV DNA: RT-PCR>8.23 log HBsAg: (+) Anti-HBsAb: (−) Anti-HBcAb total: (+) Anti-HBcAbIgM: (−) HbeAg: (−) Anti-HbeAb: (+)	AST: 670 U/L, ALT: 994 U/L	Delayed	TNF before hepatitis workup, administration high-dose steroids for potential autoimmune hepatitis	10 weeks	10 weeks
3	Koksal, 2017^7^	56 (M)	Melanoma stage IV	Ipilimumab (September 2016) after four cycles switched to Nivolumab due to AST/ALT increase	Unknown	HBsAg: (+) Anti-HBs: (−) Anti- HbcIgM: unknown HbeAg: unknown Anti-HBe: unknown	Normal	None	8 weeks (after four cycles on ipilimumab)	HBV DNA: 244.259 IU/mL HbeAg: (−) Anti-HBe: (+) Anti- HbcIgM: (+) Anti- HBs: (−) (after first cycle of nivolumab)	AST: 164 U/L, ALT: 246 U/L, after fourth cycle of ipilimumab AST: 845 U/L, ALT: 888 U/L after first cycle of nivolumab	Switched ipilimumab to nivolumab Continued nivolumab	TDF 245 mg once daily (started after the first cycle of nivolumab)	HBV DNA: 183 IU/mL, after 8 weeks of TNF	ALT/AST significantly decreased (56 U/L and 50 U/L, respectively) after 8 weeks of TNF
4	Akar, 2019^8^	62 (M)	Clear-cell RCC stage IV	Nivolumab (prior treatments: sunitinib, axitinib and RT)	Undetectable	HBsAg: (+) HDV DNA: (+) after sunitinib	Normal	Entecavir	40 weeks (18 cycles)	**No HBV reactivation** after 18 cycles of nivolumab and antiviral prophylaxis with entecavir (HBV DNA (−))	AST: 28 U/L, ALT: 19 U/L	Continued	Entecavir, since HBV/HDV reactivation under sunitinib	Negative HBV DNA after 40 weeks ofnivolumab (underentecavir)	AST 28 U/L, ALT 19 U/L, after 40 weeks of nivolumab (under entecavir)
5	Zhang, 2019^9^	48 (M)	NPC	Camrelizumab	Undetectable	HBsAg (+)	Not defined	None	3 weeks	HBV DNA: 7.81 × 10^3^	ALT: 191.4 U/L	Delayed	Entecavir	1 week	2 weeks
6	Zhang, 2019^9^	47 (M)	NPC	Camrelizumab	Undetectable	HBsAg (+)	Not defined	None	16 weeks	HBV DNA: 6.98 × 10^4^	ALT: 203.0 U/L	Delayed	Entecavir	4 weeks	4 weeks
7	Zhang, 2019^9^	39 (M)	Melanoma	Camrelizumab	Undetectable	HBsAg (+)	Not defined	None	28 weeks	HBV DNA: 2.10 × 10^3^	ALT: 27.6 U/L	Continued	None	5 weeks	N/A
8	Zhang, 2019^9^	36 (M)	HCC	Nivolumab	Undetectable	HBsAg (+)	Not defined	Entecavir	12 weeks	HBV DNA: 1.80 × 10^3^	ALT: 298 U/L	Discontinued	Entecavir + TNF	1 week	3 weeks
9	Zhang, 2019^9^	45 (M)	HNSCC	Toripalimab	Undetectable	HBsAg (+) Baseline HBV	Not defined	None	35 weeks	HBV DNA: 4.04 × 10^6^	ALT: 281.2 U/L	Delayed	Entecavir	3 weeks	6 weeks
10	Zhang, 2019^9^	41 (F)	Soft tissue sarcoma	Nivolumab	Undetectable	HBsAg (+)	Not defined	None	20 weeks	HBV DNA: 6.00 × 10^7^	ALT: 465.1 U/L	N/A	Entecavir	8 weeks	4 weeks
**A/a**	**First author, year**	**Age (gender)**	**Cancer type**	**Type of ICPI**	**Baseline** **HCV RNA**	**Baseline hepatitis profile**	**Baseline** **AST/ALT**	**Antiviral prophylaxis**	**Duration on ICPI at reactivation**	**Reactivation** **HCV RNA (IU/mL)**	**Reactivation** **AST/ALT (U/L)**	**Management of ICPI**	**Antiviral treatment**	**Time for achieving undetectable HCV RNA (weeks)**	**Time for ALT (ULN:56 U/L) and AST (ULN:40 U/L) recovery (weeks)**
1	Kothapalli, 2018 58	54 (M)	Melanoma stage IV	Pembrolizumab	HCV RNA: (+)	Hepatitis panel work-up performed after ALT elevation	Normal	None	8 weeks	Chronic HCV infection (HCV RNA (+))	Grade II ALT rise	Continued	Ledipasvir 90 mg/sofosbuvir 400 mg	Not defined	12 weeks
2	Davar, 2015 59	59 (F)	Melanoma stage IV	Pembrolizumab	HCV RNA: 2.290.867 IU/mL (genotype 1A)	Not defined	Mildly elevated AST/ALT	None	**No HCVr:** antiviral treatment after 9 cycles of pembrolizumab	HCV RNA stable after 3 cycles of pembrolizumab	Stable	Continued	Ledipasvir 90 mg/sofosbuvir 400 mg (after 9 cycles of pembrolizumab) Duration of antiviral treatment: 12 weeks	21 weeks (time of antiviral treatment)	41 weeks from pembrolizumab and 18 weeks from antiviral treatment
3	Davar, 2015^59^	47 (M)	Melanoma stage IV	IFN followed by ipilimumab followed by IL-2 and dabrafenib/trametinib followed by pembrolizumab	HCV RNA: 863 475 IU/mL (genotype 1c)	Not defined HIV viral load: undetectable (under ART treatment)	Normal	None	**No HCVr:** two cycles of pembrolizumab before PD	HCV RNA stable after 2 cycles of pembrolizumab	Stable	Discontinued due to PD	ART treatment (2 NRTIs+1 NNRTI)	–	–

ALT, alanine aminotransferase; ART, antiretroviral therapy; AST, aspartate aminotransferase; HBsAg, hepatitis B surface antigen; HBV, hepatitis B virus; HCC, hepatocellular carcinoma; HCV, hepatitis C virus; HCVr, HCV reactivation; HNSCC, head and neck squamous cell carcinoma; ICPI, Immune checkpoint inhibitor; IFN, interferon; NNRTI, non-nucleoside reverse transcriptase inhibitor; NPC, nasopharyngeal carcinoma; NRTIs, nucleoside reverse transcriptase inhibitors; NSCLC, non-small-cell lung cancer; PD, progression of disease; RCC, renal cell carcinoma; RT, radiotherapy; RT-PCR, reverse transcription polymerase chain reaction; TDF, tenofovir disoproxil fumarate; TNF, tumor necrosis factor; TTF-1, thyroid transcription factor 1.

Just two case studies describe the outcomes of nine patients with HCV infection treating with ICPIs. Davar *et al*^59^ presented two cases with metastatic melanoma: (1) the first one had a chronic HCV infection and achieved partial response on pembrolizumab with no significant elevation of AST/ALT (grade 1), and stable HCV viral load throughout immunotherapy that became undetectable after initiation of antiviral treatment, and (2 the second one had a chronic HCV/HIV coinfection and showed melanoma progression on pembrolizumab with grade 1 elevation of AST/ALT, undetectable HIV and variable HCV viral loads during immunotherapy course.^59^ A larger case series by Kothapalli and Khattak investigating safety and efficacy of PD-1 inhibitors for metastatic NSCLC or melanoma in patients with viral hepatitis presented three patients with chronic HCV infection (one with additional past HBV infection) receiving pembrolizumab or nivolumab.^58^ Only one patient experienced a grade 2 elevation of ALT, which was normalized following anti-HCV treatment with ledipasvir 90 mg/sofosbuvir 400 mg. However for the majority of patients, no details regarding viral load trends are available and these results should also be interpreted with caution.

In the clinical trials of advanced HCC where HBV-infected or HCV-infected patients were included, no hepatitis-related flares were reported. In the CheckMate-040 trial, 51 HBV-infected cases (all receiving concurrent antiviral therapy) and 50 HCV-infected patients (antiviral therapy was not required) with advanced HCC were treated with nivolumab, none of these patients experienced hepatitis reactivation.^10^ Interestingly, nivolumab exhibited limited anti-HCV activity with a transient RNA reduction but without achieving a sustained virologic response (SVR) for greater than 6 months.^10^ In KEYNOTE-224 study, 22 HBV-infected and 26 HCV-infected patients with advanced HCC were treated with pembrolizumab but no flares of hepatitis occurred.^11^ In both trials investigating anti-PD-1 treatment on HBV-related HCC, patients were required to receive antiviral therapy in order to have a viral load of <100 IU/mL at screening and were regularly monitored for HBsAg but not for detectable HBV DNA.^10 11^ In another multicenter study, 14 patients with HBV and 14 with HCV infections (the majority diagnosed with melanoma) responded to anti-PD-1/PD-L1 immunotherapy (three responses in each viral group) without unexpected toxicity or ≥1 log increase in viral load.^60^ Approaching the same issue from the opposite direction, an interesting trial by Sangro *et al*^12^ included 22 patients with chronic HCV and HCC after failure of sorafenib in firstline and examined the mixed antitumor and antiviral activity of CTLA-4 blockade with tremelimumab. Although, that some patients had a transient intense elevation of transaminases after the first dose, none of them faced exacerbation of HCV infection during the subsequent cycles; instead partial response and disease control was achieved in 17.6% and 76.4% of them, respectively.^12^ Given the too small sample sizes in the abovementioned studies, no strong conclusions could be extracted, regarding the rate and the risk factors for hepatitis reactivation as well as the necessity of antiviral prophylaxis in patients receiving ICPIs.

## Screening and prophylaxis for HBV and HCV before immunotherapy

So far, universal screening of patients with newly diagnosed cancer for HBV and HCV is not a routine in oncology practice. Initially, the majority of experts’ societies including AASLD,^61^ AGA^53^ and American Society of Clinical Oncology (ASCO)^62^ proposed a risk-adaptive HBV screening in patients at high risk for latent HBV infection (eg, birth in an endemic country, contact with HBV-infected persons, intravenous drug use or HIV coinfection) or in patients with planned anticancer therapy associated with high risk for HBVr. Screening includes both HBsAg and anti-HBc assessment, since HBVr can occur in patients with chronic infection, HBsAg(+)/anti-HBc(+) or in those with clinically resolved infection, HBsAg(−)/anti-HBc(+). The role of anti-HBs is not been established yet but its presence may decrease the HBVr risk.^63^ In patients with HBsAg(−)/anti-HBc(+), anti-HBs may be useful for detecting past infection, and may predict HBVr when diminish in surveillance.^57 64 65^

In cases with HBsAg(+)/anti-HBc(+) (eg, chronic HBV infection), antiviral pre-emptive therapy should be started as soon as possible before (ie, most often 7 days) or, at the latest, simultaneously with the onset of immunosuppressive therapy using anti-HBV drugs with a high resistance barrier, such as entecavir, TDF, or tenofovir alafenamide (TAF) and should be continued during therapy and for at least 6 months (or for at least 12 months in patients receiving anti-CD20 antibodies and up to 18 months according to EASL) after its completion.^33 66^ Three preliminary trials of patients with HBsAg(+)/anti-HBc(+) receiving anticancer therapy had supported the preventive use of lamivudine,^49 67 68^ but latter studies showed the superiority of entecavir over lamivudine.^53 69 70^ Since late reactivations (ie, beyond 12 months) have been also reported,^71 72^ patients should be long-term followed up after antiviral prophylaxis cessation.

In patients with HBsAg(−)/anti-HBc(+) (eg, clinically resolved HBV infection), the risk of HBVr varies widely according to their virological profile, their underlying disease and their immunosuppressive schema (eg, type and duration). Moreover, the strategy is depending on their clinical situation and feasibility of close monitoring (ie, ALT, HBsAg and/or HBV DNA every 1–3 months during and after immunosuppression) with the intent of on-demand antivirals at the first sign of HBVr (pre-emptive therapy), except for patients receiving anti-CD20 treatment or undergoing SCT, for whom prophylaxis is recommended.^32 33 62^ The EASL Clinical Practice Guidelines suggest that all candidates with HBsAg(−)/anti-HBc(+) for immunosuppressive therapy should be tested for serum HBV DNA before immunosuppression and if it is detectable, they should be treated with anti-HBV prophylaxis, similarly to patients with HBsAg(+). In agreement with EASL, the updated AASLD guidance,^32^ the CDCP recommendations and the alternative proposal by ASCO consensus support a universal testing for HBsAg and anti-HBc in all patients before initiation of any anticancer treatment. In a meta-analysis of studies with patients with HBsAg(−)/anti-HBc(+) receiving immunosuppressive therapy, the pooled HBVr rate was estimated relatively high (6.5%) and anti-HBV prophylaxis was recommended in hematological malignancies and/or rituximab-containing regimens, regardless of HBV DNA and anti-HBs status.^63^ Instead, patients with solid tumors or rituximab-free regimens were reported to have a low risk of HBVr and may not require prophylaxis if they have undetectable viral load and anti-HBs(+).^63^

In response to previously reported data, we agree that immunotherapy should be included in this universal screening strategy and antiviral prophylaxis should be recommended in patients with viremia with detectable serum HBV DNA at baseline. Putting cost-effectiveness parameters in our considerations for universal screening, local HBV prevalence should also be taken into account, particularly in countries with too low HBV rates. In these countries, screening should be followed only for patients at high risk for HBV infection (citizens in endemic areas, drug abusers, HIV coinfection, etc) or for patients anticipating high-risk treatments for HBVr (SCT or chimeric antigen receptor T cells (CAR-T) or (non)-myeloablative chemotherapy). However, the optimal management of patients with HBsAg(−)/anti-HBc(+), the threshold of HBV DNA levels to start prophylaxis, the most appropriate nucleotide analogs (NAs) in terms of efficacy and cost trade-off, and the recommended duration of preventive treatment need to be determined. Given the durable effect of immunotherapy beyond its administration period and the general late event of HBVr, antiviral therapy should be continued for at least 6 months after the last dose of ICPI and not be stopped even after HBV DNA negativity.

For HCV, current guidelines support universal screening in patients with hematologic malignancies and hematopoietic SCT recipients,^73 74^ but there is no optimal strategy for patients with non-hepatic solid cancers. According to an observational study from MD Anderson Cancer Center, only 13.9% of patients with cancer were screened for HCV infection.^75^ The initial screening for HCV infection is based on the serum detection of anti-HCV antibodies. As previously mentioned, a positive anti-HCV test could not differentiate acute from resolved infection (after spontaneous viral clearance or antiviral therapy) or a false positive result. When testing for anti-HCV is positive, a PCR assay for HCV RNA quantification must be performed, together with clinical and laboratory examination.^76^ In order to overcome the high level of unawareness for HCV status in newly diagnosed patients with cancer^2^ and the sustained false-negative rate of selective screening,^77^ all patients with cancer should be screened for HCV infection.^25^

## Antiviral therapy

When HBV DNA monitoring at-risk patients without prophylaxis demonstrates reactivation, the preferred antivirals for deferred (‘on-demand’) treatment are NAs.^54^ Available NAs including lamivudine, entecavir, adefovir, tenofovir, TAF, and telbivudine produce a potent suppression of viral replication but are associated with a low rate of HBsAg serological clearance and a high risk of viral relapse after discontinuation. Because of these reasons, long-term treatment with NAs is needed to maintain virologic response but durable administration is feasible, well tolerated and without major side effects. Lamivudine, the first used NA, found to have higher resistance rate and thus its administration waned in cases treated with long-term systemic regimens.

In a direct comparison between NAs, entecavir has been found to be more effective (and more expensive) than lamivudine.^53 69 70^ This superiority has been proved by a recent randomized controlled trial (RCT) where HBV-associated hepatitis rates in the entecavir group were significantly lower compared with that in lamivudine group (0% vs 13.3%; p<0.003).^70^ The AASLD and EASL agree that patients with HBsAg(+)/anti-HBc(+) should receive entecavir or TDF or TAF as treatment or prophylaxis while subjects with HBsAg(−)/anti-HBc(+) should receive anti-HBV prophylaxis if they are candidates to receive high HBVr immunosuppressive treatment.^32 33^ At the end, the selection among available NAs should be a shared decision of both oncology providers and hepatology experts and the whole comanagement of patients with cancer with chronic or clinically resolved HBV infection should be overviewed at a multidisciplinary level.^78^ Noteworthy, when NAs are usually administered as on-demand treatment to attenuate liver injury and improve patient outcomes, but these results are significantly better when NAs are used in advance as prophylaxis. Indeed, data from observational studies suggest that the overall rate of HBVr is considerably lower when prophylactic antiviral therapy is compared with deferred treatment with NAs.^69^ However, most of these studies are of poor quality, use heterogeneous definitions of HBVr, report inconsistently their outcomes, and monitor HBV DNA levels following different time points and methodologies.

Regarding the management of chronic HCV infection in patients with cancer, the updated guidelines by AASLD and Infectious Diseases Society of America (IDSA) without robust evidence from RCTs supported that the overall benefits of DAAs in terms of virologic, hepatic, and oncologic outcomes far outweigh the risks of not treating.^25 77 79^ Bearing in mind the contraindications, including pregnancy, short life expectancy (eg, <12 months), known hypersensitivity to DAAs or potential drug–drug interactions, all patients with cancer with chronic HCV infection should be treated with DAAs without significant delay.^77 80^ In view of efficacy, a large cohort of 141 HCV-infected patients with any type of malignancy received sofosbuvir-based therapy and achieved at 12 weeks a SVR rate of 91%.^81^ In confirmation, smaller studies in patients with hematologic malignancies replicated SVR rates at 12 weeks of 98%–100% with interferon-free DAAs treatment.^82 83^ The quick eradication of chronic HCV infection by DAAs offers multiple clinical profits: help liver recovery, diminish the risk of HCVr, allow patients to receive enhanced immunosuppressive anticancer therapies and to access into oncological clinical trials, and finally reduce the risk of developing HCV-associated hepatic and extrahepatic cancers. SVR found to be associated with a 71%–76% reduction in HCC risk compared with the risk of not achieving,^84 85^ while a recent meta-analyis demonstrates also a clear association of SVR with a better outcome of HCV-positive B-NHL (OR=9.34, 95% CI: 4.90 to 17.79, p<0.00001).^86^

In HCV-infected patients with cancer, the administration of newer DAAs, such as of glecaprevir/pibrentasvir for 8 weeks or sofosbuvir/velpatasvir for 12 weeks is proposed as a feasible and effective strategy as suggested to patients without cancer.^77^ This short duration of DAA treatment facilitates fast completion of anti-HCV therapy before or between cycles of anticancer treatment, avoiding concomitant administration, which may be associated with adverse effects and overlapping toxic effects.^25^ Here, we should definitely admit that the use of DAAs in HCV-infected patients with cancer receiving concomitantly oncological treatments hides also an increased risk of drug–drug interactions.^25 77^ These drug–drug interactions are usually predicted studying drug pharmacokinetics, metabolism and clearance, since safety and surveillance data are not enough, especially for the recently approved regimens (eg, glecaprevir/pibrentasvir, sofosbuvir/velpatasvir ± voxilaprevir). In the single prospective study investigating the safety of DAAs given concomitantly with chemotherapy or biological agents, no drug–drug interactions were reported and selected anti-HCV regimen was altered in only three patients.^87^ When cancer treatment could not be interrupted, newer DAAs should be simultaneously administered, after ruling out any potential interaction. As recommend in patients without cancer, DAA treatment should be offered under close co-monitoring by medical oncologists and hepatology experts while larger studies are warranted to optimize this approach.^79 81^

## Discussion

Current data regarding efficacy and safety of ICPIs in patients with HBV and HCV are derived from case reports, case series, retrospective cohort studies and few clinical trials on advanced HCC, leading to a very low evidence level. Immunotherapy seems to be associated with no inferior efficacy in cases with previous or chronic HBV/HCV infections, nor with major safety issues regarding antiviral or immunologic response. Despite the well-tolerated profile of ICPIs in these patients, the behavior of viral hepatitis under ICPI is scarcely investigated and probably underestimated in the literature. The existing evidence on hepatitis–PD1 interface could not estimate the exact reactivation risk induced by ICPIs and thus, could suggest but not sufficiently support a universal hepatitis screening. However, the management of immune-related hepatotoxicity is coming to agree with a comprehensive baseline assessment of liver status, including screening for prior HBV/HCV infections.^88^ According to ESMO and ASCO guidelines, liver function tests at the onset and before every immunotherapy cycle, will help the early diagnosis of immune-related hepatitis while the diagnostic work-up for all contributory reasons of liver injury including viral infection, autoimmune reaction or alcohol/drug consumption should be performed before the initiation of an ICPI and repeated, whenever aminotransferases rise, with or without concurrent bilirubin elevation.^14 88 89^ Patients with underlying liver diseases should be monitored more closely and be earlier referred for hepatology consultancy, even from the initiation of immunotherapy.

Even if baseline viral hepatitis screening could be modeled as a cost-effective strategy in patients with cancer, as already demonstrated in the general population,^32 90 91^ the answer is not so clear for the treating approach. The on-demand treatment at the time of hepatitis reactivation appeared to have poorer response compared with the pre-emptive use of modern antiviral drugs.^77^ The preventive therapy could eliminate or diminish up front the viral load in most cases, leading to cure or long-term hepatitis control.^77^ Despite the efficacy of antiviral prophylaxis, the therapeutic decision for underlying hepatitis and the whole oncological management should be supported by a multidisciplinary team, after a thorough discussion of the potential benefits, risks and costs. Given the high prevalence of HBV/HCV infections in populations with cancer and without cancer, a large number of patients should be pre-emptively treated in order to avoid a relevantly low reactivation risk. Numerically, the extra cost of prophylaxis with generic NAs in screened and selected HBV-infected patients with cancer is minor over the already high financial burden of immunotherapy.^32 91^ However, the issue becomes more complex in the setting of HCV, due to the high cost of modern anti-HCV medications. Although AASLD and IDSA do not use cost-effectiveness analysis to guide their recommendations, the cost of DAAs, either in preventive or in deferred treatment, continues to limit their public health impact.^79^ Pharmaceutical competitions and government negotiations try to bring prices to the point where all persons in need of anti-HCV treatment are able to access it. However, all these financial considerations end up on cancer prognosis. In HCV-infected cases receiving curative or palliative treatment with estimated long-term survival, the appropriate antiviral prophylaxis can subsequently decrease the direct costs of averting hepatitis-related complications and the overall healthcare budget by preventing later primary or secondary hepatitis-associated malignancies.^92^ Instead, in cases with limited survival, the administration of expensive DAAs for 12 weeks, with only prophylactic intent, must be considered as a futile and less reasonable option.

Navigating into uncharted water, we should notice that further prospective immunotherapy trials including hepatitis-infected patients with cancer are required in order to strengthen the suggested management. There is still a lot to learn about the way that ICPIs affect the immune vigilance of underlying HBV and HCV infections, while numerous parameters are under consideration in the final therapeutic decision for both cancer and viral diseases, including the viral course, the nature of metastatic cancer, the immune-mediated mechanisms of administered treatments, the expected patient response and prognosis, as well as the financial costs of selected therapies. Treating this special patient population represents a clinical challenge in everyday oncological practice and still, a shift is needed in the design of modern trials to reflect more representatively real-world scenarios and to enable more precise extrapolation of research findings.
